# Management of deceased and living kidney donor with lithiasis: a multicenter retrospective study on behalf of the renal transplant group of the Spanish urological association

**DOI:** 10.1007/s40620-024-01960-5

**Published:** 2024-06-22

**Authors:** Alba Sierra, Begoña Etcheverry, Mario Alvarez-Maestro, Juan Manuel López, Maria Fiol, Carlos Torrecilla, Francesc Vigués, Carmen Martínez, Enric Carbonell, Salvador Martinez-Perez, Antonio Alcaraz, Maria Pilar Luque, Mireia Musquera

**Affiliations:** 1 Department of Urology, Hospital Clinic–University of Barcelona, Barcelona, Spain; 2 Department of Urology, Hospital de Bellvitge–University of Barcelona, Barcelona, Spain; 3 Department of Urology, Hospital Universitario La Paz, Madrid, Spain; 4 Departament de Cirurgia i Especialitats Médicoquirúrgiques, Universitat de Barcelona, Barcelona, Spain; 5 Servei Urología, Hospital clínic, Barcelona, Spain

**Keywords:** Kidney transplant, Urolithiasis, Flexible ureterorenoscopy (f-URS), Conservative treatment, Back table endoscopy

## Abstract

**Background:**

To maximize the availability of suitable grafts and ensure effective management, several reports have demonstrated successful outcomes when using kidney grafts with urolithiasis. This multicenter study reports on the management and long-term outcomes of kidney transplantation using renal grafts with lithiasis.

**Methods:**

Retrospective data from three Spanish hospitals were analyzed for kidney transplants involving grafts with nephrolithiasis performed between December 2009 and August 2023. The study included adult patients, excluding those with incomplete records. It evaluated stone characteristics, complications, and outcomes in recipients and in living kidney donors.

**Results:**

Out of 38 analyzed kidney transplants, 57.9% were cadaveric and 42.1% were from living kidney donors. Most diagnoses were incidental during donor evaluation, with an average stone size of 7.06 mm. After follow-up (median 26 months), all recipients but one had functioning grafts, and there were no stone recurrences in both recipients and living kidney donors. Conservative management was adopted in 28 cases, while 10 cases required *ex-vivo* flexible ureterorenoscopy for stone removal. Following conservative management, 5 patients needed additional treatments for stone-related events.

**Conclusions:**

Kidneys with lithiasis can be considered for transplantation in selected cases, resulting in good functional outcomes with no stone recurrence in recipients or living donors.

**Graphical Abstract:**

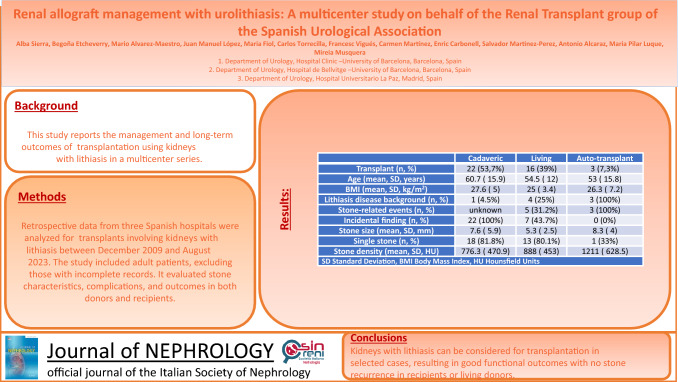

## Introduction

Numerous studies have demonstrated the superiority of kidney transplantation over dialysis for patients with end-stage kidney disease, leading to improved survival and enhanced quality of life [1]. To maximize the availability of suitable grafts and ensure effective management, several reports have demonstrated successful outcomes when using kidneys with urolithiasis [2–4], the estimated prevalence of which is 0.64%. However, the incidence of nephrolithiasis has notably increased over the past three decades, likely due to changes in the environment and dietary habits, which led us to consider these grafts to increase the donor pool [5, 6].

The Amsterdam Forum on the Care of the Live Kidney Donor delineated specific acceptance criteria for asymptomatic potential donors with a history of a single stone, including (1) absence of hypercalciuria, hyperuricemia, or metabolic acidosis; (2) no cystinuria or hyperoxaluria; (3) no urinary tract infection; and (4) no evidence of multiple stones or nephrocalcinosis on computed tomography (CT) [7].

As in all cases of solitary kidneys, immediate intervention is mandatory to minimize the potential risks of obstruction, sepsis, and graft dysfunction due to urinary stone obstruction [8]. Therefore, it is crucial to establish appropriate management strategies for these non-standard risk donors. To date, given the limited literature on the subject, shared criteria for the surgical management of kidney graft stones have yet to be established.

In this study, we present our results of the management and long-term follow-up in a multicenter series involving kidneys with kidney stones managed conservatively or surgically for stone removal prior to kidney transplant.

## Materials and methods

### Patient selection

Three high-volume and experienced kidney transplant centers (Hospital Bellvitge—Hospitalet, Spain; Hospital La Paz—Madrid, Spain; Hospital Clinic—Barcelona, Spain) participated in this retrospective review of electronic medical records and kidney transplant databases to collect data regarding patients who underwent kidney transplantation with lithiasis. Data were collected and analyzed to assess the prevalence, characteristics, and outcomes of kidney transplants with nephrolithiasis. When urolithiasis was identified in the CT or ultrasound, donor comorbidities and stone characteristics were recorded. In addition, all living kidney donor candidates were asked about stone-related events, and a basic metabolic workup was performed. Patients with incomplete medical records or missing data were excluded from the analysis.

### Management and interventions

The observation and treatment modalities employed for lithiasis management, including medical treatment (alkalinization), extracorporeal shock wave lithotripsy (ESWL), and *ex-vivo* flexible ureterorenoscopy (f-URS) were recorded.

Regarding flexible ureterorenoscopy for graft stone removal, after cold perfusion in back table endoscopy, with the kidney still immersed in ice slush, a retrograde flexible ureterorenoscopy was performed as described previously [4]. If this technique failed, pyelolithotomy was performed to remove the stone.

### Data collection

Data were retrospectively collected, including stone volume, Hounsfield units (HU), and kidney stone location. When back table endoscopy was performed for stone removal, both cold and warm ischemia times were collected from surgery reports. Stone composition was collected from Fourier-transform infrared spectroscopy and morphological analysis reports. Postoperative complications were evaluated according to the Clavien-Dindo classification and long-term follow-up was assessed to evaluate kidney function and urinary stone recurrence in both living kidney donors and recipients.

### Statistical analysis

Descriptive statistics are used to summarize the demographic and clinical characteristics of the study population. Continuous variables are presented as medians with interquartile ranges. Categorical variables are expressed as percentages.

## Results

The study included 38 kidney transplants, with 26, 7, and 5 performed at Hospital Bellvitge, Hospital Clinic, and Hospital La Paz, respectively. Of these, 22 (57.9%) were obtained from deceased kidney donors (DKDs) and 16 (42.1%) were from living kidney donors. Donors had a mean age of 56 (± 14.6) years, while recipients had a mean age of 57.1 (± 15.5) years. The average Body Mass Index (BMI) for donors and recipients was 26.3 (± 5.2) and 26.2 (± 4.9) kg/m^2^, respectively. Among the living kidney donors, 81.8% were blood relatives of the recipients, 25% had a history of urolithiasis, while in the remaining cases the diagnosis was incidentally made during donor evaluation using CT. No prior medical reports were available for deceased kidney donors (Table 1).

**Table 1:** Demographics of donor population and stone characteristics

	Cadaveric	Living
Transplant (*n*, %)	22 (57.9%)	16 (42.1%)
Age (mean, SD, years)	60.7 (±15.9)	54.5 (±12)
BMI (mean, SD, kg/m^2^)	27.6 (±5)	25 (±3.4)
Lithiasis disease background (*n*, %)	1 (4.5%)	4 (25%)
Stone-related events (*n*, %)	Unknown	5 (31.2%)
Incidental finding (*n*, %)	22 (100%)	7 (43.7%)
Stone size (mean, SD, mm)	7.6 (±5.9)	5.3 (±2.5)
Single stone (*n*, %)	18 (81.8%)	13 (80.1%)
Stone density (mean, SD, HU)	776.3 (±470.9)	888 (±453)
Conservative	22 (100%)	6 (37.5%)
Bench surgery	0	10 (62.5%)

*SD* standard deviation, *BMI* body mass index, *HU* hounsfield units

A single kidney stone was identified in 81.6% of cases, with a mean stone density of 832.3 (± 462.5) Hounsfield units and a mean stone size of 6.45 (± 4.1) mm. Notably, one living kidney donor with a 13 mm stone underwent extracorporeal shock wave lithotripsy three months pre-transplant, resulting in the removal of small residual stones measuring 4 and 2 mm in the lower calyx during back table endoscopy. Of the 10 patients who underwent back table endoscopy for graft stone removal, only two cases with single 8 mm stones failed to progress to the proximal ureter and required pyelolithotomy. In this group, no stone-related events were reported, and after stone removal no stone recurrence was observed during the follow-up (Table 2). Of note, no stone recurrence was observed in the contralateral kidney among the living kidney donors.

**Table 2:** Demographics of recipient population and surgical outcomes

Transplant	Age (Years)	Sex	BMI	Comorbidities	Type of transplant	Stone size (mm)	Stone removal technique	Ischemia Time (min)		Delayed Graft Function	Stone characteristics	Postoperative complications	Stone-related events	1st-week Cr (mg/dl)	Nadir Cr (mg/dl)	Follow-up (months)	Kidney function (Cr. mg/dl)	GFR (ml/min/l)
								Cold	Warm									
1	63	M	24.7	HTA. duodenal ulcer. diverticulitis	LD	6	f-URS	46	2	No	Whewellite 95% (Randall plaque)	Paralytic ileus	No	2.31	1.12	47.7	1.18	64
2	45	M	24.8	None	LD	4	f-URS	43	1.37	No	Whewellite 90% Carbapatite 5% Weddellite 5%	Arterial thrombosis (7 days after)						
3	45	F	21.3	HTA. chronic anemia	LD	8	f-URS + Pyelothomy	41	3	No	Carbapatite 80% Whewellite 10% Proteins 10%	No	No	2.55	1.99	42.5	2.43	23
4	34	F	18.8	HTA	LD	6	f-URS	38	3	No	Weddellite 70% Whewellite 20%	Wall hematoma	No	2.78	0.54	40.2	1.49	45
5	56	M	26	Ischemic heart disease	LD	13	SWL + f-URS	45	1.18	Yes	Whewellite 80% Uric acid 20%	No	No	8.92	1.23	149.5	1.44	50
6	66	F	18	HTA. DM-I. sarcoidosis	LD	5.33	f-URS	37	2.4	No	Whewellite 95%	No	No	1.1	1.07	8.8	1.31	57
7	63	F	22.9	HTA. DM- 2. dyslipidemia. eosinophilic cystitis	LD	3	f-URS	44	2.3	No	Uric acid > 95%	No	No	1.57	1.03	4.5	1.22	47
8	39	F	25	Chronic inflammatory bowel disease	LD	7.2	f-URS + Pyelothomy	69	3	No	Whewellite 90% + calcium phosphate 10%	No	No	1.53	1.06	37.2	1.53	44
9	54	M	26	IgA nephropathy. HTA. hyperuricemia	LD	5	f-URS	47	2	No	Unknown	No	No	2.97	1.33	19.2	1.38	54
10	46	M	19	None	LD	5	f-URS	43	2	No	Whewellite 30%. Weddellite 30% Calcium phosphate 20% Struvite 20%	No	No	2.4	1.64	1	1.64	50

*M* male, *F* female, *BMI* body mass index, *HTA* hypertension, *DM* diabetes mellitus, *LD* living donor, *f-URS* flexible ureterorrenoscopy

Among conservatively managed grafts, mean stone size was 7.7 (± 4.8) mm. There was a significant increase in stone size in two cases (< 1 mm/year) and both remained asymptomatic. Stone-related events occurred in five cases, with treatments including shock wave lithotripsy, percutaneous nephrolithotomy (PCNL), and flexible ureterorenoscopy. In one case, acute obstructive pyelonephritis occurred, thus an attempt was made to remove the stone via flexible ureterorenoscopy, which, unfortunately, was unsuccessful and the patient developed ureteral stenosis, requiring open ureteral reimplantation 16 months after kidney transplant. Despite these challenges, the graft still maintains good kidney function after six years (Table 3).

**Table 3:** Demographics of recipient population after conservative treatment

Transplant	Age (Years)	Sex	BMI	Type of transplant	Stone size (mm)	Comorbidities	Ischemia Time (min)		Delayed Graft Function	Immediate postoperative complications	Stone-related events	Cr (1st week)	Nadir Cr (mg/dl)	Follow-up (months)	Kidneyfunction (Cr, mg/dl)	GFR (ml/min/l)
							Cold	Warm								
1	35	M	26.4	LD	5	HTA. Hyperuricemia	120	4.5	No	Partial kidney infarction		3.34	1.2	162.7	1.6	55
2	65	M	27.9	DKD	7	Ischemic heart disease. HTA IgA nephropathy	108	38	Yes	No	Acute pyelonephritis (5 months)	9.88	1.14	80.6	1.5	52
3	34	F	24.9	DKD	5	HTA bilateral vesicoureteral reflux	670	50	No	Bleeding		3.7	0.87	17.3	1.1	55
4	27	M	31.2	DKD	8	HTA. Alport syndrome	553	84	Yes	No		7.65	0.98	13.6	1.5	66
5	64	M	31.1	DKD	10	HTA. DM. vascular disease	450	58	No	Femoral arterial ischemia		2.45	1.13	5	1.4	54
6	50	F	22	LD	4	SLE	21	4	No	No	Stone size increase	1.91	0.98	83.2	1.1	59
7	75	M	28	LD	5	HTA	62	2	No	No		3.01	1.15	121.2	1.3	52
8	65	M	25	DKD	12	Auricular fibrillation. DM. HTA. cerebrovascular accident	1131		No	No		2.46	1.46	71.2	1.7	55
9	73	M	31	DKD	6.5	HTA. DM. obesity atrial fibrillation	1205		Yes	No		12.01	1.02	67.2	2.5	24
10	24	M	22	LD	5	Myocardiopathy thrombotic microangiopathy	102	6	No	No		2.95	1.32	50.8	2.8	30
11	58	F	23	LD	3	Schoenlein Henoch. HTA. dyslipidemia	37	3	No	No		4.3	0.9	37.9	1.1	55
12	56	M	30	DKD	8	Obesity. DM. cerebrovascular disease	28	6	No	No	SWL	2.74	0.59	36.8	1.4	58
13	68	M	28	DKD	8	DM. neurogenic bladder	16		No	No		2.36	0.8	32.6	0.8	75
14	73	M	23	DKD	9	*None*	1128		Yes	No		Dialysis	0.5	2	0.5	> 90
15	48	F	34	DKD	7	*None*	1389		No	No		2.05	0.5	4.7	0.5	84
16	49	M	24	DKD	24	HTA. obesity	907		No	No		1.2	1.22	25.9	1.3	64
17	73	F	24	DKD	17	HTA. DM	1097		Yes	No		5.43	0.95	25.6	1.5	35
18	56	F	21	DKD	2	HTA. DM. dyslipidemia severe aortic stenosis	1062		No	No		2.6	1.38	23.2	1.7	40
19	81	F	29	DKD	4	HTA	1440		No	No	Stone size increase	1.6	1.07	17.6	1.2	42
20	30	F	20	DKD	7	*None*	1183		Yes	No	f-URS	Dialysis	1.6	18.3	1.6	43
21	60	M	32	DKD	2	*None*	343	12	Yes	Bleeding		7.32	1.19	16.9	1.2	64
22	58	M	40	DKD	7	HTA	351	15	No	No	PCNL	3.53	1.19	15.7	1.6	41
23	61	M	24	DKD	8	Myocardiopathy. HTA. DM. VHC	775		Yes	No		6.4	0.8	9.6	1.4	53
24	77	M	32	DKD	5	HTA. DM. obesity. prostate cancer	294		No	Bleeding		3.33	1.3	8.2	1.3	55
25	72	M	31	DKD	8	HTA. obesity. OSAS. DM	1320		Yes	Bleeding		10.88	1.02	6.2	2.2	28
26	82	M	24	DKD	16	DM	1337		Yes	No	PCNL	8.59	1.06	3	1.4	46
27	70	F	32	LD	3	HTA. breast cancer	37	2	No	No		1.67	0.98	1.7	1.3	46
28	77	M	30	DKD	10	HTA. DM	1038	28	No	No		3.04	1.13	1.2	1.9	33

*M* male, *F* female, *BMI* body mass index, *LD* living donor, *DKD* deceased kidney donor, *HTA* hypertension, *SLE* systemic lupus erythematosus, *DM* diabetes mellitus, *SWL* shock wave lithotripsy, *f-URS* flexible ureterorrenoscopy, *PCNL* percutaneous nephrolithotomy

Kidney stone analysis was performed after stone removal, and most calculi were found to contain whewellite and weddellite as the main components (69.2%) (Table 2). Among the living kidney donors, the mean cold ischemia time was 45.3 (±8.9) minutes in the surgical group and 63.17 (±39.7) min for the conservative group, while in the deceased kidney donor group the mean cold ischemia time was 14.2 (±7.4) hours. As regards living kidney donor transplants, one patient experienced delayed graft function, after the kidney was submitted to the two-stage stone treatment. During a follow-up of twelve years, the kidney graft recipient maintains good kidney function with creatinine levels of 1.44 mg/dl and is stone-free. Among the deceased kidney donor transplants, ten patients presented delayed graft function; all underwent conservative stone management.

Immediate intraoperative bleeding occurred in four cases in the conservative group, which was attributed to the vascular anastomosis procedure in one patient, to capsular bleeding in two others, while in the fourth patient bleeding was linked to the removal of a 4.5 cm angiomyolipoma during back table endoscopy (Table 3). Other postoperative complications in the latter patient included paralytic ileus, transplant arterial thrombosis and femoral arterial ischemia. This patient passed away five months later due to influenza-related complications with a functioning kidney graft. After a median follow-up of 26 (range: 1–163) months, grafts had a mean creatinine level of 1.39 (± 0.5) mg/dl and a GFR of 52.3 (± 15.35) ml/min/l (Tables 2 and 3).

## Discussion

In the past, renal lithiasis was considered an absolute contraindication for donation. However, this is a now manageable condition, thanks to recent advances in kidney stone treatment [3, 9, 10]. Our multicenter study included a cohort of kidney transplant recipients who received allografts with kidney stones and had long-term follow-up. The therapeutic decision was based on stone size and the transplant center committee’s evaluation at each hospital, as shared criteria for the surgical management of stones in kidney grafts have yet to be established. There was no specific management of kidney stones from living kidney donors.

Among the 10 recipients of a kidney which underwent back table endoscopy, all stones were successfully extracted prior to kidney transplantation, resulting in a graft survival rate of 90%. In the group of 28 patients for whom observation was chosen, none displayed signs of graft impairment. However, seven patients experienced stone-related events during follow-up. Moreover, in the living kidney donor transplants, longer cold-ischemia time was observed in the conservative group, meaning that back table endoscopy does not increase the surgical time if well planned.

While most series report outcomes and treatments for stone formation after kidney transplantation, few studies focus on lithiasis in the kidney graft [4, 11]. The lack of comprehensive literature on stone management in transplanted kidneys has led to ongoing debates regarding the most effective approaches. Currently, options include observation, extracorporeal shock wave lithotripsy, endourologic interventions, percutaneous nephrolithotomy, and open surgery [3, 10].

The largest series, reported by Jan MY et al., involved 57 donor-recipient pairs managed conservatively; the median stone size was of 2 (range: 1–6) mm, with a similar follow-up (3.5 years) but worse graft survival (78.9%). None of the negative outcomes were stone-related [11]. In our study, 66.7% of patients underwent observation and five were subsequently surgically treated due to obstructive complications. Conservative management is recommended for single stones smaller than 4 mm [5]. It should be noted that, in these patients, stones were mostly located in the medium calyx and they presented multiple small stones; thus, perhaps we should consider selecting patients with a lower number of calculi to reduce the risk of complications as, in other studies, a high failure rate for spontaneous stone passage in such cases was reported [12, 13]. Nevertheless, larger but single stones located in the lower calyx exhibited better results. In our series, stones up to 10 mm located in the lower calyx underwent conservative management without problems. However, several factors have been associated with graft lithiasis progression, such as urinary stasis, reflux, recurrent urinary tract infections, renal tubular acidosis, pH changes, and supersaturated urine [14]. Addressing these factors before a kidney transplant may significantly contribute to successful outcomes. Moreover, a strict follow-up of these patients to anticipate complications is essential.

On the other hand, flexible ureterorenoscopy during back table endoscopy allowed effective management of stones measuring 4–10 mm, while larger stones required supplementary treatments. Urolithiasis in kidney transplant recipients often presents asymptomatically due to graft denervation, posing challenges in early detection. According to our results, managing stones before transplantation is essential to prevent stone-related complications post-transplantation. Moreover, most of the available series regarding grafts with lithiasis report the need for post-transplant surgical treatments [12, 13]. Thus, it is reasonable to proactively treat urinary calculi prior to transplantation.

Urinary stones measuring between 10 and 15 mm pose a greater challenge, therefore, the European Association of Urology promotes extracorporeal shock wave lithotripsy and endourology in this situation, as grade B recommendations [15]. In our study, a patient with a 13 mm stone received a two-stage treatment. Moreover, two stones of around 8 mm were not extracted by flexible ureterorenoscopy and pyelolithotomy was needed. It would be advisable to identify in advance organs in which stone extraction is not feasible, to reduce cold ischemia time.

To guide personalized management, basic metabolic stone screening, measuring urinary calcium, oxalate, uric acid, creatinine, pH, etc., is recommended [15]. In our cohort, only one living kidney donor presented metabolic alteration at evaluation. Unfortunately, deceased kidney donors cannot be metabolically evaluated. Once removed, analysis of the stone is mandatory to study its etiology and prevent recurrences [15, 16].

Given the retrospective nature of our study, we acknowledge its limitations, and in particular the low number of cases studied. Conducting large prospective studies remains challenging due to the low prevalence of kidney stones in kidney donors. Our findings highlight the efficacy and safety of minimally invasive procedures in managing incidental renal lithiasis in kidney donors. None of the living donors in our series presented stone recurrence in their remaining kidney during the follow-up period.

## Conclusions

Dealing with kidney grafts with lithiasis is uncommon and the decision to use such organs for transplantation raises doubts due to the limited evidence available. Adequate counseling and diligent monitoring are crucial for both donors and recipients. The therapeutic choices vary according to stone size and location; however, to reduce stone-related events after transplantation, ex-vivo flexible ureterorenoscopy can be performed with good graft outcomes.

## Data Availability

All data generated or analyzed during this study are included in this published article.
